# Antimicrobial Resistance & Migrants in Sweden: Poor Living Conditions Enforced by Migration Control Policies as a Risk Factor for Optimal Public Health Management

**DOI:** 10.3389/fpubh.2021.642983

**Published:** 2021-07-01

**Authors:** Mangrio Elisabeth, Paul-Satyaseela Maneesh, Sjögren Forss Katarina, Zdravkovic Slobodan, Strange Michael

**Affiliations:** ^1^Department of Care Science, Faculty of Health and Society, Malmö University, Malmö, Sweden; ^2^Malmö Institute for Studies of Migration, Diversity and Welfare (MIM), Malmö University, Malmö, Sweden; ^3^R&D Directorate, Acharya Institute of Technology, Bengaluru, India; ^4^Global Political Studies, Malmö University, Malmö, Sweden

**Keywords:** antimicrobial resistance, health assessment, health policy, migration, precarity

## Abstract

Infectious diseases exacerbated by Antimicrobial Resistance (AMR) are of increasing concern in Sweden, with multi-drug resistant strains associated with new resistance mechanisms that are emerging and spreading worldwide. Existing research has identified that sub-optimal living conditions and poor access to healthcare are significant factors in the spread and incubation of AMR strains. The article considers this linkage and the effort to control the spread of AMR in relation to migrants, highlighting deficiencies in public policy where such individuals are often increasingly exposed to those conditions that exacerbate AMR. In many of the richest countries, those conditions are not accidental, but often direct goals of policies designed with the goal of deterring migrants from staying within host countries. Without engaging with the politics around migration control, the article points to urgent need for more holistic assessment of all public policies that may, however unintentionally, undermine AMR control through worsening living conditions for vulnerable groups. The consequences of prioritizing policies meant to deliberately worsen the living conditions of migrants over avoiding those conditions that accelerate AMR spread, are today made ever apparent where new AMR strains have the potential to dwarf the societal effects of the current Covid-19 pandemic.

## Introduction

Infectious diseases exacerbated by Antimicrobial Resistance (AMR) are of increasing concern, with multi-drug resistant strains associated with new resistance mechanisms that are emerging and spreading worldwide. The re-emerging pattern of AMR spread both among humans and animals is of major concern, and its impact in terms of costs for individuals and society. According to the Surveillance atlas of infectious diseases database, AMR is rising in several European countries (1). In this paper we consider the paradoxical situation in which the need to combat the spread of AMR is potentially undermined by policies seen worldwide that expose a growing population in many countries to worsening health and living conditions. Worsening living conditions for marginalized populations, made vulnerable on the grounds of their legal status as irregular migrants, create the conditions for AMR to spread and develop (2, 3). We further aim to address how this issue could be dealt with in Sweden, in order to improve health for migrants as well as protection for rest of the population. The article is structured by first outlining the health vulnerability experienced by individuals subject to forced migration and deteriorating conditions within host countries. Second, we consider the role health vulnerabilities play in the development of AMR. Consequently, we argue, that the health vulnerability imposed on a growing group of marginalized migrants needs to be addressed as a threat to policies mitigating AMR. In the current paper we include refugees, asylum seekers and undocumented migrants within the term migrants.

### Refugee Health and Health Care Needs

Forced migration is known to cause physical & psychological stress, due to the circumstances that surround both feeling from their country of origin, and applying for asylum within the host country (2). Migrants suffer from social stress through problems with finances, discrimination, as well as that of learning a new language, and culture (3). A Swedish research project (4, 5) studied recently arrived migrants during 2018 and 2019 as well as those being in the country for several years. The research showed that recently arrived migrants had good self-reported health, but began to experience challenges including mental ill health, allergies, overweight, obesity, physical inactivity, lack of social relations, and support (4, 5). The research also showed that every second newly arrived migrant had been in need of health-care during the first 3 months, but could not use it due to the above challenges (4, 5). These unmet healthcare needs are more common among the newly arrived migrants than the refugees who have lived in the country for a longer period (4, 5). Further research within the MILSA platform in Sweden showed that the newly arrived migrants seem to be unsatisfied with the health-care, and also experienced difficulties in navigating the system regarding referrals and access to care (6). A possible explanation might be limited health literacy (7), i.e., the abilities people have to gain and understand basic health information and instructions in the medical context (esp. by learning a new language) as well as services that they need to make proper decisions about their health (8, 9). Health literacy among newly arrived migrants has been found to be low (10) and related to an overall poor health, not seeking healthcare even if needed (7). Furthermore, where migrants are subject to enhanced migration controls, the psychological impact of managing the demands imposed, forces them often to down-prioritize their health needs over caring for family, or managing their precarious legal status (11).

### Health Assessment at the Refugee Health Clinics

The newly arrived migrants in Sweden receive information regarding practical issues, including health-care on arrival. They are encouraged to approach the refugee health clinic established in every town; this information is available on the web page “New in Sweden” (12). Adult asylum seekers in Sweden have the right to obtain a health assessment as well as health-care and dental-care that could not be deferred. They also have the right to preventive health-care during pregnancy as well as contraceptive counseling. Asylum seeking children have the right to obtain the same health- and dental-care as resident children of Sweden (13). Research among migrants in Sweden showed that around 65% of all newly arrived migrants used the option of having a health assessment at the refugee health clinic (10); but with fewer men compared to women. New-published research about the health assessments for migrants in Sweden, shows that the migrants perceive difficulties with getting proper information about the purpose and aim of the assessments, and often are suspicious that the visit will negatively affect their asylum application (14). Since the Refugee Health Clinic is responsible for all the newly arrived migrants for their health-care access until they receive permission to stay, there is a need to explore and identify the barriers they face in obtaining the health-care to which they have a formal right (10). There is also a need to investigate the needed improvements in obtaining health-care that meets the needs of this group in society. Of growing importance in this context is the extent to which refugees are made vulnerable to AMR through lacking, or feeling unable to, access healthcare, as will be discussed next.

### Antimicrobial Resistance and Health

Today, antibiotic therapy is threatened by bacterial resistance that is emerging faster than we can discover and develop new drugs (15). This is untenable and leads to severe consequences such as morbidity and mortality; it is likened to the next pandemic (16). Bacterial pathogens continuously develop resistance to antibacterial agents that are used clinically as an evolutionary process contributing to the challenge of AMR. It complicates the treatment for bacterial infections, which results in concurrent use of multiple antibiotics, prolonged treatments and long hospitalizations. Worldwide this is an emerging challenge, since it is estimated that every year around 700,000 people die of infections caused by Multi-drug resistant (MDR) strains. The resistance is becoming a crisis since the antibiotic pipeline is weak, lacking new antibiotics needed to stay ahead of the emerging resistance (15). Among migrants, infectious diseases exacerbated by AMR is of particular concern since MDR strains are associated with new resistance mechanisms that are emerging and spreading worldwide (17). Migrants are potentially exposed to multiple strains of AMR like all mobile populations including tourists, but are at particular risk due to crowded and inadequate conditions in refugee camps and Holding Centers, extreme psychological stress due to the conditions of precarity they experience, and often lack access to medical care for conditions that are otherwise easily treatable (17).

A WHO report on the health of migrants in the Europe states that AMR is increasing worldwide and that forced migration and discriminatory policies in host countries may play a role by worsening living conditions (18). Although, surveillance for AMR in the WHO European Region is among the most advanced in the world, there are limited data available on the role of displacement and migration in Europe. But existing evidence suggests that migrants are exposed to conditions that are favorable for the development of AMR (18). Whilst there is little data regarding the extent migrants carry AMR bacterial strains, what evidence there is indicates that migrants fleeing the Syrian crisis are vulnerable to pathogens due to often lacking access to clean water and sanitation, and being exposed to crowded living conditions, within conflict zones but also European host countries (20, 21). A recently published review of research on AMR amongst migrants in Europe (19), found that the prevalence of any carriage or infection with AMR organisms was higher amongst those subject to forced migration and the exclusionary living conditions that often follow, than in other migrant groups. The prevalence of AMR organisms was slightly higher in community settings with large numbers of migrants, such as refugee camps and detention facilities, than in hospital settings. It is important to clarify, the review did not find evidence of high rates of transmission of AMR from migrants to host populations (19). Migrants are vulnerable to AMR due to the conditions linked to their precarity, including those experienced in host countries, but do not pose a risk to host populations. An Australian systematic review focusing on migrants and AMR concludes with a statement that further studies on AMR and migrants should concentrate on establishing AMR databases by collecting samples from AMR cases among the migrants as well as incorporate new policies and guidelines to improve the detection, prevention and control of AMR among migrants (17). There is also clear need for more research on the role inadequate conditions for receiving migrants, as well as policies deliberately intended to worsen their life conditions, play in the ongoing development of AMR. The review of research on AMR and migrants in Europe, by Nellums et al. (19), identifies the need for improved living conditions, access to health care and initiatives to facilitate detection of and appropriate high-quality treatment for AMR infections during transit and in host countries, and that protocols to prevent and control AMR should include measures to address the challenges faced by migrants (19).

AMR is not just a threat for health but carries economic burdens for society. For example, according to an estimate by the Swedish Public Health Agency, by 2050 the number of cases will be four times more than today an cost society approximately 16 billion Swedish Kroner (1.9 billion US dollars) (20). Other estimates warn that a continued rise in resistance by 2050 would lead to 10 million people dying every year and a reduction of 2–3.5% in Gross Domestic Product (GDP), costing globally up to 100 trillion USD (21). Much of the literature places the blame for this potential scenario on continuing poor antimicrobial stewardship (22, 23).

## Discussion

In order to support the newly arrived migrants in their health-care needs on arrival, several measures need to be taken. Many may have succumbed to various medical conditions (incl. exposure to AMR) during transit before arriving in the country, and are at risk of worsening conditions, if not appropriately attended to at the earliest moment. We stress that governing authorities must be able to reassure the newly arrived migrants that health assessments are made immediately and remain confidential with no bearing on their eligibility for the right to stay. Failure to provide such reassurance would risk that those policies intended to protect public health will most likely prove counterproductive by only exacerbating the deteriorating conditions experienced by migrants that favor the incubation of AMR. In addition to health assessments, it is necessary to screen for infectious diseases immediately on arrival and extend prophylactic measures at an earlier stage. If the newly arrived migrants are infected, then this measure could benefit both - the migrants, as well as the host population, by better controlling and preventing the spread of AMR.

There are examples of infection control at the time of arrival amongst migrants (24, 25), but not much published yet on screening for AMR at borders of entrance into the host countries. Although, based on a systematic review of what research exists, de Smalen et al. (17) suggest that compulsory AMR screening of migrants should be incorporated as a first preventive measure. It has been suggested that the emphasis around screening for infectious diseases among migrants must be placed on developing innovative and sustainable strategies to facilitate the screening and to improve treatment completion (25). Implementing compulsory screening alone is unlikely to be of benefit, due to the conceivably high risk that it would drive those most at risk to find ways to avoid medical assistance as well as posing ethical problems.

In addition to health screening to mitigate AMR, we also need to better understand the impact of current migration control policies on the life conditions that may exacerbate the incubation of AMR. Migrant populations are subject to levels of control not experienced by the rest of the population, with likely considerable effects on their vulnerability to AMR. Without reliable baseline data on AMR amongst different populations, it is not possible to directly measure the role migration control policies have on AMR, but there is however substantial data linking those policies to worsening life conditions which are understood as risk factors in AMR (11, 26–30). For example, migration control policies in the UK that require healthcare professionals to assess an individual's migration status prior to receiving treatment, as well as the introduction of fees for some, has created significant uncertainty amongst both those professionals and refugees and asylum seekers such that many have either been refused or not sought healthcare despite their need and entitlement (31). Whilst refugees and asylum seekers formally have access, there is significant confusion amongst both health professionals and those requiring healthcare that undermine the legal entitlement. The capacity of migrants to manage their own healthcare, including seeking medical assistance, needs to be understood within the context of the life conditions they experience with respect to also their legal status as either asylum-seekers, those granted refugee status, or those whose cases have been rejected and are living in an irregular situation. Policies designed to meet political goals of migration control that negatively impact the welfare of migrants need to be assessed in terms of public health criteria particularly where there is a risk that such restrictions are exacerbating the development of AMR. Providing appropriate health and social care to refugees is an issue that goes beyond the welfare of those individuals to concern public health, with broader economic and security implications. Whilst policies that stigmatize migrants may suit the political tastes of the time, it should be clear – particularly in the context of Covid-19, – that those policies come at a high cost if they, whether deliberately or not, exclude vulnerable groups from healthcare and expose them to conditions conducive to AMR pathogens spread.

There is some evidence that new technologies may help increase access to healthcare for marginalized groups, including migrants. According to a systematic review on health literacy in the eHealth era, mobile apps have a great potential to deliver interactive health services custom-made to people with low health literacy (32). Such tools need to be approached carefully, given that marginalized migrants often lack direct access to a mobile phone, with a single phone often shared between multiple and non-familial individuals with practical and privacy issues that often prevent access to digital technologies on a reliable basis. However, in incorporated within a wider series of tools that includes non-digital means for accessing health information, soft-tools such as mobile applications can sometimes help newly arrived migrants navigate their health-care visits as well as access basic information on the health care system available since it is easier to both update and, importantly, translate information. This aids in improving health as well as the health care access for these people, and also provides a platform for understanding the development of AMR in the context of the global shift toward migration controls that have significantly impacted the life conditions of people lacking stable residency. Developing a more holistic model of AMR in which life conditions are seen to play a significant role, health literacy also requires that wider society is better educated on the health negativities that follow from current migration control policies. That is not to enter into a debate on the political merits of migration control, but to better understand the societal and economic negativities that may follow from peripheral policies that lead to, e.g., lower access to health services and increasingly crowded and inappropriate accommodation.

In conclusion, early access to health-care and health equity among the migrants appear to be critical. If AMR is not tackled within health-care settings, the healthcare risks being ineffective in reaching its goals of good health for all (33). Whilst there is no evidence that migrants can transmit AMR to host populations (22), there is a very real risk that – as seen with Covid-19 – that science is ignored by some in favor of a politically popular rhetoric in which migrants are blamed. What we see in the existing research is a clear sign that such attitudes may well-proved detrimental to not only such vulnerable groups, but the whole society. Where an increasing part of the world's population have become forced migrants, legitimizing policies that undermine their healthcare creates the ideal conditions necessary to incubate future strains of AMR pathogens with the potential to spread globally despite migration controls. The commitment to ensure health-care must be provided to assist human populations in distress and be followed by concerted actions of various agencies engaged in the process. Improved living conditions and access to healthcare should be essential goals, relevant to migrants, and all vulnerable groups, for the sake of not only their welfare but to reduce the likelihood of new AMR strains capable of much greater societal negativities than seen with the current Covid-19 global pandemic. There is a bitter irony here, in that many of those policies that have worsened the living conditions of migrants are sold *via* a rhetoric of “national security,” but that in the case of AMR now pose a very real security threat to all national societies unless their negative impact on human lives can be reversed.

## Data Availability Statement

The original contributions presented in the study are included in the article/supplementary material, further inquiries can be directed to the corresponding author/s.

## Author Contributions

ME, P-SM, and SM took the lead in writing this paper and have given an equal contribution to the work with the paper. SK and ZS has read the paper and given comments and suggestions on the writing. All authors have read and approved the last version of the paper.

## Conflict of Interest

The authors declare that the research was conducted in the absence of any commercial or financial relationships that could be construed as a potential conflict of interest.
